# Predictors of Successful Quitting among Thai Adult Smokers: Evidence from ITC-SEA (Thailand) Survey

**DOI:** 10.3390/ijerph121012095

**Published:** 2015-09-25

**Authors:** Aree Jampaklay, Ron Borland, Hua-Hie Yong, Buppha Sirirassamee, Omid Fotuhi, Geoffrey T. Fong

**Affiliations:** 1 Institute for Population and Social Research, Mahidol University, Salaya, Nakhon Pathom 73170, Thailand; 2 Cancer Council Victoria, Melbourne, Victoria 3004, Australia; E-Mails: ron.borland@cancervic.org.au (R.B.); hua.yong@cancervic.org.au (H.-H.Y.); 3 Institute for Population and Social Research, Mahidol University, Salaya, Nakhon Pathom 73170, Thailand; E-Mail: buppha.sir@mahidol.ac.th; 4 Department of Psychology, Stanford University, 450 Serra Mall, Stanford, CA 94305, USA; E-Mail: omidfotuhi@gmail.com; 5 Department of Psychology, University of Waterloo, Waterloo, ON N2L 3G1, Canada; E-Mail: geoffrey.fong@uwaterloo.ca; 6 School of Public health and Health Systems, University of Waterloo, Waterloo, ON N2L 3G1, Canada; 7 Ontario Institute for Cancer Research, Toronto, ON M5G 0A3, Canada

**Keywords:** predictors of successful quitting, adult smokers in Thailand, ITC surveys

## Abstract

This study uses longitudinal data from the International Tobacco Control Southeast Asia (ITC-SEA Thailand) survey to explore patterns and predictors of successful quitting among Thai adult smokers as a function of time quit. A cohort of a representative sample of 2000 smokers was surveyed four times from 2005 to 2009. A sample of 1533 individuals provided data for at least one of the reported analyses. Over the four years of follow-up, 97% made attempts to quit. Outcomes were successful quitting/relapse: (a) quit attempts of at least one month (short-term relapse, 43%) (57% remaining quit); (b) surviving at least six months (medium-term) (31%); (c) relapse between one and six months (45%); (d) having continuously quit between Waves 3 and 4 (sustained abstinence) (14%); and (e) relapse from six months on (44%) compared to those who continuously quit between Waves 3 and 4 (56%). Predictors for early relapse (<1 month) differ from longer-term relapse. Age was associated with reduced relapse over all three periods, and was much stronger for longer periods of abstinence. Cigarette consumption predicted relapse for short and medium terms. Self-assessed addiction was predictive of early relapse, but reversed to predict abstinence beyond six months. Previous quit history of more than one week was predictive of early abstinence, but became unrelated subsequently. Self-efficacy was strongly predictive of abstinence in the first month but was associated with relapse thereafter. Some determinants of relapse change with time quit, but this may be in somewhat different to patterns found in the West.

## 1. Introduction

The aim of this paper is to better understand the process of successfully quitting smoking and how determinants of relapse might vary with time quit. It uses data from a developing country, Thailand, which has a very different history of tobacco control and a very different culture to the Western countries where most existing research on quitting emanates from. Any or all of cultural factors, level of economic development, and past efforts to control tobacco use could influence successful quitting. However, this paper focuses on individual characteristics that might predict abstinence.

Quitting smoking is a difficult process and usually involves multiple attempts [1], with a high relapse occurring, not just in the early days of an attempt, but also months after quitting [2]. We need to understand whether research from a Western context can apply elsewhere in the world. This requires information from longitudinal studies in non-Western countries. The International Tobacco Control Policy Evaluation project Southeast Asia (ITC-SEA) survey—which includes questions on a wide range of known and hypothesized mediators of smoking cessation—provides the ideal vehicle for considering predictors of quitting.

Past studies from Western countries suggest that quitting smoking has two major components: initiating an attempt and maintaining cessation once quit [3]. Hyland *et al.*’s study [4] in four developed countries (Australia, Canada, the UK, and the US) found that intention to quit, making a quit attempt in the previous year, longer duration of past quit attempts, less nicotine dependence, more negative attitudes about smoking, and younger age are predictive of making a quit attempt. However, the main factor that predicted abstinence was lower levels of nicotine dependence as indexed by the Heaviness of Smoking Index (HSI) [5]. In other studies, predictors of successful quitting vary, including older age [6,7,8], being male [6,8], higher socioeconomic status [9], lower level of nicotine dependence [10], longer length of past quit attempt [11], self-efficacy [12], motivation to quit [6], and absence of other smokers at home [6,8].

Studies on smoking cessation in Asia, including Thailand, especially longitudinal studies, are limited. In Thailand, the prevalence of smoking has declined among men aged 15 and over from 56% in 1991 to 41.7% in 2011. Among women, the change has been from 4.6% to 2.1% [13].

A recent study using the cross-sectional Global Adult Tobacco Surveys (GATS) [14] in low-to-middle-income countries, including Thailand, indicates that cigarette prices affect smoking behavior through different mechanisms. While higher cigarette prices prevent initiation in low to low-middle income countries where smoking rates are relatively lower, it increases cessation in high-middle to high income countries, where smoking rates are relatively higher. In Kostova *et al.*’s study, Thailand is classified as one among high-middle to high income countries, thus a high cigarette price may be expected to affect quitting. Using the same data set, Shang *et al.* [15] investigated the association between demographic and policy-relevant factors and the probability of being a recent quitter. Results reveal that the odds of being a quitter are associated with exposure to worksite smoking bans, exposure to anti-smoking media messaging, warning labels, cigarette prices, and bidi prices. More specifically, they found a particularly strong association between work-site smoking bans and quitting in Southeast Asian countries, including Thailand. Our study focuses on the role of endogenous factors, some of which are likely to mediate the impact of these exogenous factors.

Li *et al.* [16] explored predictors of making attempts and short-term success among those who tried to quit, based on smoking status at follow-up, using data from the first two ITC-SEA survey waves in Malaysia and Thailand. They found, in both countries, that predictors of quit attempts and remaining quit at the follow-up survey include having smoked fewer cigarettes per day, higher level of self-efficacy, and more immediate quitting intention. Previous shorter quit attempts (*i.e.*, less than six months) and higher health concerns about smoking were only predictive of making quit attempts, while prior abstinence for at least six months and older age were associated with remaining quit at the follow-up. The authors commented that predictors found in Thailand and Malaysia differed in potentially important ways from those found in the West, and that these differences are likely a mix of effects due to the earlier stage of tackling the tobacco problem in Asia and cultural differences. Some of the differences they found were socio-demographic factors, particularly when the outcome was making a quit attempt. In Thailand and Malaysia, older smokers were more likely to make quitting attempts, whereas the reverse was true in the West (see [4]). This study also found effects of majority/minority ethnic group and urban/rural residence, but no such effects were found in the West. In addition, short previous attempts were predictive of trying to quit in the ITC-SEA study, but longer previous attempts predicted trying to quit in the West. Moreover, while intention to quit was a strong positive predictor of attempts in the West, it shows only a weak effect in the SEA study.

The data for this study were gathered in a context where Thailand had launched its first mass media public education campaign in late 2005, after the baseline survey we conducted, but before the second wave of the survey. However, before that, Thailand had taken other strong measures to fight the tobacco epidemic in the region and was compliant with most of the requirements of the World Health Organization’s (WHO) Framework Convention on Tobacco Control (FCTC). In 1992, Thailand implemented a Tobacco Control Act, which, among other restrictions, includes increasing the price of cigarettes, banning tobacco advertising, banning display of cigarettes at point of sale, requiring health warning labels on cigarette pack, and limiting smoking in public places. Thailand, in 2005 (again after our baseline survey), became the fourth country in the world to implement a law requiring the use of pictorial health warning labels on cigarette packs after Canada, Brazil, and Singapore. In short, there was a marked increase in efforts to encourage quitting over the period since the baseline survey that built on a generally negative view of smoking.

There is now clear evidence to separate making quit attempts from successful abstinence among those trying [3,17]. Among those making quit attempts, there is increasing evidence that predictors of success might also vary with duration of quitting [2,18], so it is important to see if determinants of relapse differ with time. Herd *et al.* [2], found curvilinear relationships with a range of variables, including number of smoking friends (assessed before the attempt) and frequency of strong urges to smoke (assessed while quit) and subsequent relapse. Yong *et al.* [18] found that the HSI [5] was strongly predictive of relapse within the first month of a quit attempt, but among those quit for a month, was no longer predictive. This research suggests that there may be quite different determinants of short-term and longer-term relapse.

This paper expands on the analysis by Li *et al.* [16] of predictors of quitting among Thai smokers, to explore determinants of short, medium, and longer-term relapse from quit attempts to explore whether predictors of relapse differ as the quit attempt gets longer.

## 2. Experimental Section

### 2.1. Data Source and Study Sample

Data come from Waves 1–4 of the longitudinal ITC-SEA Thailand survey. Wave 1 data were in early-2005, between January and February, Wave 2 was collected between August and September, 2006, Wave 3 between January and March, 2008, and Wave 4 between April and June, 2009. A detailed description of ITC-SEA can be found elsewhere (e.g., [19]). The project received Institutional Review Board (IRB) approval by the Institute for Population and Social Research, Mahidol University Institutional Review Board with the reference number of Ref. 0517.191/0705 on 28 June 2007. Briefly, the ITC-SEA Thailand survey, conducted by the Institute for Population and Social Research at Mahidol University, is the first longitudinal study of a nationally representative sample of adult smokers in Thailand recruited via cluster sampling and face-to-face interviews. It is one of the few population-based cohort studies of smoking cessation. The original sample in Wave 1 consisted of 2000 smokers aged 18 and older. We followed up with all those we could; including re-contacting those lost at previous waves and retained ex-smokers. The original sample included 9% women (172 cases) and of this original sample, 1533 provided data on at least one outcome. Among this sample, 44 respondents had never tried to quit during Wave 1 to Wave 4, so we excluded them in our analysis. Thus, the analytical sample is 1489.

### 2.2. Measures and Analysis

Our main outcome of interest is relapse in relation to successful quitting, for which we explored 5 outcome measures based on different criteria as defined below among those who reported any quit attempts over the course of the study:

1. *Short-term relapse*, defined having relapsed within 1 month on all quit attempts between Waves 1 and 4.
2. *Medium-term quitting*, defined as having quit for at least 6 months over the same interval among all those who made attempts.
3. *Medium-term relapse*, which was relapsing between 1 and 6 months after starting *versus* remaining quit for longer (the same success criterion as in outcome 2).
4. *Sustained abstinence*, defined as being quit continuously from before Wave 3 to Wave 4 (*i.e.*, without relapsing) among all who made attempts.
5. *Longer-term relapse*, defined relapsing after 6 months and, thus, not achieving the sustained abstinence criterion (cessation outcome 4).

The choice of cut-points for duration of abstinence is partly arbitrary, but is consistent with many other studies, which treat 1 month as short-term and greater than 6 months as the criterion for longer-term abstinence [20,21,22].

For the purpose of maximizing the power for detecting an effect, cases that were lost in a particular wave but recovered in other waves and had met one or more criteria (*i.e.*, 1 month or 6 months abstinence) were included for analysis. The recovered cases account for about 15% of the original total sample (300 out of 2000).

Please note that the information on the length of quit at subsequent waves after Wave 1 to meet the criteria of each outcome considers only a consecutive, not cumulative length. For example, for Outcome 1, a smoker is considered a short-term relapser (coded 0) if s/he failed to achieve 1 months of abstinence on any attempt, but if they did quit for at least a month on any occasion between waves 1 and 4, they were coded as a quitter (Outcome 1 = 1).

Our independent variables were all measured at Wave 1 and are primarily measures of individual beliefs and experiences. Guided by Li *et al.* [16], the main covariates selected were measures of nicotine dependence, past quit attempts, intention to quit (not plan to quit, plan to quit but in more than six months, plan to quit in the next six months, and plan to quit within the next month), and self-efficacy. There were three indices of nicotine dependence: number of cigarettes smoked per day, time to first cigarette (after breakfast *versus* before breakfast), and perceived addiction (very addicted, somewhat addicted, and not addicted). Note that the measure of time to first cigarette, using events (*i.e.*, breakfast) was developed for this study based on advice we received, that rural Thais were unlikely to be able to answer time to first cigarette in minutes and hours. Perceived addiction has been used previously in ITC surveys and other places [23]. Our interest was whether the smokers overall perception of their level of addiction was predictive. The correlation among these three indicators show that they are not highly correlated (*i.e.*, the correlation coefficients (*r*) are between 0.27–0.38). We also measured quitting history using longest length of past quit attempts at the baseline wave.

Self-efficacy was measured based on how sure the respondents were that they would be successful if they were to quit (coded as “not at all sure” *versus* “somewhat sure”, “very sure”, and “extremely sure”). We also coded whether the smoker smoked predominantly factory-made, predominantly hand-rolled cigarettes, or both. Information on type of cigarette may well reflect the effect of cigarettes price as found significant on smoking behavior in previous study, e.g., [14]. In Thailand, the price of factory-made is much higher than hand-rolled cigarettes. As data on the price of cigarettes is not available in our data set, we use information of type of cigarette to proxy the price of cigarettes. Type of cigarette may also reflect the perception of Thai smokers on the health impact of smoking. Previous research indicates that more than one fourth of Thai smokers (29%) perceive that hand-rolled cigarettes are more harmful than factory-made cigarette, while almost one-third (31%) think the other way around [24].

Measures of smokers’ perception about quitting and smoking were also included as followed. Perceived benefit of quitting is a dichotomous variable classified as “very much benefit” *versus* “otherwise” which includes “somewhat benefit” and “not at all benefit”. Level of worry about health is classified as “somewhat”, “very much”, and “not at all”. Enjoyment of smoking is dichotomized as “agree/strongly agree” *versus* “strongly disagree/disagree/neither agree nor disagree” that they enjoy smoking too much to quit. Finally, smoking ban at home is also a dichotomous variable categorized as “smoking not allowed anywhere at home” *versus* “allow some place/no restriction”.

Socio-demographic factors included respondent’s sex, age group, educational level, marital status, and region of residence.

We employed logistic regression models to explore predictors of our 5 measures of successful quitting. Five sets of the analyses were undertaken, using each outcome criterion separately. We used the same set of independent variables, entered as one block, for all analyses (see Table 3 for the list).

## 3. Results and Discussion

Nearly all (97%) smokers made at least one attempt over the study period. Table 1 presents percentages achieving each of the five quitting/relapse outcomes among those making quit attempts along with sizes of the sample for each analysis. Of those making quit attempts, 57.3% survived on one or more attempt for at least one month, 31.2% lasted at least six months and 14.1% managed to stay quit across the last two follow-up waves (at least). This meant that of those quit for one month, 45.4% relapsed by six months, and, of the survivors at six months, 43.8% subsequently relapsed leaving only 56.2% who managed to stay quit at least for the last two waves.

**Table 1:** Summary of the five sets of regressions on predictors of quit success relapse among those making quit attempts over the period of the study.

Regression Model among Those Making Quit Attempts	Criterion for Successful Quitting, Coded “1”		Criterion for Relapse, Coded “0”		Excluded from Analysis
		N		N	
1. Short-term success *vs.* short term relapse (within 1 month)	Made one or more quit attempts that lasted at least one month duration between waves 1 and 4	853	Quit attempts which all lasted less than a month	636	None
2. Medium-term success: Quitting for 6 months or more among all who made quit attempts	Made one or more attempts that lasted at least 6 months	340	Quit attempts, which all lasted for less than 6 months	749	None
3. Medium-term relapse (between 1 and 6 months)	Made one or more quit attempts that lasted at least six months	340	Made attempts that lasted at least 1 month, but less than 6 months	283	Those making quit attempts of less than 1 month
4. Sustained abstinence (*i.e.*, well over 1 year)	Remained quit for the last two waves of the study (at least)	147	Made quit attempts but did not remain quit over the period Wave 3 to Wave 4	899	None
5. Long term relapse: (beyond 6 months)	Remained quit for the last two waves of the study (at least)	146	Made quit attempts that lasted at least 6 months, except for those who did not relapse after Wave 3	114	Those making quit attempts of less than 6 months

NB: All analyses were restricted to those who made quit attempts over the period of the study, thus excluding the 44 cases who did not report an attempt.

**Table 2:** Characteristics of the analytical sample (both smokers and those quit for 6 months at W4), compared with the sample lost to follow-up.

Characteristics		Analytical Sample			W1 Sample Who Lost to Follow up in Subsequent Wave(s)
		Overall	Quit for more than 1 Month between W1–W4	Always Relapsed within a Month	
*N*		*1489*	*853*	*636*	*467*
Male		92.4	93.2	91.4	92.1
Age					
18–29		9.6	7.3	12.7	27.2
30–44		29.9	29.1	31.0	35.1
45–54		30.6	30.4	30.8	18.4
55–64		18.1	19.2	16.7	9.2
65+		11.8	14.1	8.8	10.1
Education					
<Primary		8.7	9.4	7.9	6.4
Primary		67.5	68.8	65.7	52.3
Secondary		16.7	15.2	18.7	29.6
>Secondary		7.1	6.6	7.7	11.8
Region of residence					
Bangkok		5.4	4.6	6.5	26.8
Urban		20.6	22.2	18.6	18.6
Rural		74.0	73.3	75.0	54.6
Amount of cigarette in a day					
≤5		22.2	25.1	18.4	23.1
6–14		37.9	38.5	37.1	38.1
15+		39.9	36.5	44.5	38.8
Have first cigarette after breakfast		37.3	39.7	34.1	31.3
Addiction to cigarette ^(n = 461)^					
Not addicted		12.3	14.9	8.8	12.2
Somewhat addicted		52.8	54.9	50.0	51.2
Very addicted		34.9	30.3	41.2	36.7
Plan to quit					
Not plan to quit		57.1	53.0	62.6	66.0
Not within next 6 months		20.0	19.3	20.8	17.8
Within next 6 months		15.2	17.8	11.6	10.7
Within next month		7.8	9.9	5.0	5.6
Self-efficacy ^(n = 455)^					
Not at all sure		35.4	27.1	46.5	40.0
Somewhat sure		36.3	38.1	33.8	35.0
Very sure		18.1	21.3	13.7	16.3
Extremely sure		10.3	13.5	6.0	8.8
Type of cigarette					
Factory-made only		41.6	41.7	41.4	59.5
Hand-rolled only		34.0	34.8	32.9	22.5
Both		24.5	23.5	25.8	18.0
Longest quit attempt at Wave 1					
	Never	22.0	17.8	27.7	24.6
	≤1 week	28.3	21.5	37.4	34.7
	>1 week–	36.2	42.4	27.8	30.6
	6 months+	13.5	18.3	7.1	10.1
Very much benefit if quit ^(n = 453)^		84.5	85.1	83.7	84.3
Worries about health ^(n = 455)^					
	Not at all	7.8	7.5	8.2	9.5
	Somewhat	35.7	32.9	39.5	40.4
	Very much	56.5	59.6	52.4	50.1
Enjoy smoking too much ^(n = 458)^		37.3	36.2	38.7	45.0
Smoking ban at home ^(n = 466)^		10.8	12.5	8.5	14.6

Table 2 shows the descriptive characteristics of our analytical sample measured at the baseline survey by whether they survived for one month or not, and for those lost to follow-up. Drop-outs tended to be younger, have higher education, live in Bangkok, and smoke factory-made cigarette. Otherwise, they were not much different from those included in our analysis.

Smokers at Wave 1 who survived at least one month (compared with early relapsers) tended to be older, smoke less, have their first cigarette later in the day, report being less addicted, more likely to plan to quit within six months, more likely to report very sure/extremely sure to successfully quit, more likely to ever quit for six months or more before Wave 1, and more likely to have a smoking ban at home.

Table 3 presents the results of the predictive analyses. The critical columns to look at in interpreting the analyses are columns 1, 3 and 5, because they relate to independent sets of relapse periods: early—within one month, medium—between one and six months, and longer term—beyond six months, respectively. Columns 2 and 4 provide information about how combining relapses across periods affects the results, and, thus, provides overall effects for the entire period. Age was a strong independent predictor of quitting regardless of quit duration. Being aged 30 and older was associated with increased success, in both the short and long terms, with the size of effects increasing for the longer intervals, particularly for abstinence beyond six months in those aged 45–54.

Higher number of cigarettes smoked per day was associated with greater relapse, although the effect is not significant for relapse beyond six months. It is notable that our novel measure of time to first cigarette was not predictive.

Having previously quit for more than one week predicted short-term success, but especially having previously quit for six months was no-longer significantly predictive for sustained quitting beyond six months.

**Table 3:** Predictors of successful quitting behaviors for each outcome measure.

Predictors		Quit for more than 1 Month *vs.* Relapse				Quit at W2–4 for 6 Months or more *vs.* Relapsed within 6 Months				Relapsed between 1 and 6 Months				Quit at both W3–4 *vs.* Relapsed Earlier no Relapse				Relapsed after 6 Months Quit, so not Still Quit between W3–4			
		Odds ratio	95%CI			Odds ratio	95%CI			Odds ratio	95%CI			Odds ratio	95%CI			Odds ratio	95%CI		
Male		1.5	0.9	2.3		1.0	0.6	1.6		0.7	0.3	1.3		0.5	0.3	1.0	*	0.7	0.3	1.7	
Age group (ref: <30)																					
	30–44	1.7	1.1	2.6	*	2.4	1.3	4.6	**	1.8	0.8	4.0		4.1	0.9	18.4		8.8	1.0	80.7	
	45–54	1.7	1.1	2.5	*	2.2	1.1	4.1	*	1.8	0.8	3.9		7.4	1.7	32.5	**	23.1	2.5	209.7	**
	55–64	2.0	1.2	3.2	**	4.1	2.0	8.1	***	3.4	1.5	7.9	**	10.8	2.4	47.9	**	16.3	1.8	148.0	*
	65+	3.4	2.0	5.9	***	5.9	2.8	12.3	***	3.7	1.5	9.4	**	10.4	2.2	48.0	**	10.4	1.1	97.4	*
Education (ref: <Primary)																					
	Primary	1.0	0.7	1.5		1.3	0.8	2.2		1.5	0.8	2.8		1.9	1.0	3.6		1.4	0.5	3.9	
	Secondary	0.8	0.5	1.3		1.3	0.7	2.4		1.6	0.7	3.5		1.4	0.6	3.3		1.3	0.3	5.1	
	>Secondary	0.8	0.4	1.4		1.0	0.4	2.2		1.3	0.5	3.6		1.3	0.4	4.0		1.1	0.2	5.5	
Region of residence (ref: Bangkok)																					
	Urban	1.3	0.8	2.3		1.2	0.6	2.4		1.0	0.4	2.6		4.0	0.9	18.1		3.2	0.5	22.4	
	Rural	1.0	0.6	1.7		0.9	0.4	1.7		0.9	0.4	2.2		3.2	0.7	14.1		2.3	0.4	15.1	
Amount of cigarette smoke (ref: ≤5)																					
	6–14	0.8	0.6	1.2		0.6	0.4	0.9	**	0.6	0.4	0.9	*	0.7	0.4	1.2		0.7	0.3	1.4	
	15+	0.8	0.6	1.1		0.5	0.3	0.7	**	0.5	0.3	0.8	**	0.5	0.3	1.0	*	0.7	0.3	1.6	
Have first cigarette after breakfast		1.0	0.7	1.2		1.2	0.9	1.6		1.4	1.0	2.1		1.1	0.7	1.7		0.9	0.5	1.7	
Level of addiction (ref: Not addicted)																					
	Somewhat addicted	0.9	0.6	1.3		0.6	0.4	1.0	*	0.6	0.4	1.1		1.0	0.6	1.7		1.2	0.6	2.6	
	Very addicted	0.7	0.5	1.1		0.6	0.3	0.9	*	0.7	0.4	1.3		1.3	0.7	2.4		2.7	1.1	6.8	*
Plan to quit (ref: not plan to quit)																					
	Not within next 6 months	0.9	0.6	1.2		0.9	0.6	1.3		0.8	0.5	1.3		0.7	0.4	1.2		0.7	0.3	1.6	
	Within next 6 months	1.2	0.9	1.8		1.2	0.8	1.8		0.9	0.6	1.5		1.2	0.7	2.0		1.0	0.4	2.4	
	Within next month	1.4	0.8	2.2		1.9	1.1	3.4	*	1.9	0.9	3.9		1.1	0.6	2.2		0.5	0.2	1.4	
Self-efficacy (ref: Not at all sure)																					
	Somewhat sure	1.8	1.4	2.4	***	0.9	0.6	1.3		0.5	0.3	0.8	**	0.8	0.5	1.3		0.4	0.2	1.0	*
	Very sure	2.0	1.4	2.9	***	1.3	0.9	2.1		0.8	0.5	1.4		1.6	0.9	2.7		0.8	0.3	1.8	
	Extremely sure	2.4	1.5	3.9	***	1.7	1.0	2.9		0.9	0.4	1.8		1.8	0.9	3.6		0.9	0.3	2.5	
Type of cigarette (ref: Factory)																					
	Hand-rolled	0.7	0.5	1.0	*	0.7	0.5	1.0		0.8	0.5	1.2		0.9	0.5	1.4		1.3	0.7	2.7	
	Both	0.7	0.5	1.0	*	0.7	0.5	1.0	*	0.8	0.5	1.3		0.9	0.5	1.5		1.0	0.4	2.1	
Longest time of quit (ref: Never)																					
	≤1 week	0.9	0.7	1.2		1.1	0.7	1.7		1.3	0.7	2.3		1.3	0.7	2.3		1.3	0.5	3.4	
	>1 week–	2.2	1.6	2.9	***	1.6	1.1	2.4	*	1.0	0.6	1.7		1.8	1.1	3.1	*	1.4	0.6	3.3	
	6 months+	3.6	2.4	5.4	***	2.9	1.8	4.7	***	1.8	1.0	3.3		1.8	1.0	3.5		0.7	0.3	1.8	
Very much benefit if quit		0.9	0.6	1.2		1.0	0.7	1.6		1.3	0.8	2.1		0.9	0.5	1.6		0.7	0.3	1.8	
Worries about health (ref: Not at all)		0.9	0.6	1.5																	
	Somewhat	1.1	0.7	1.7		0.9	0.5	1.7		1.0	0.5	2.0		0.9	0.5	2.0		1.5	0.5	4.2	
	Very much	1.2	0.9	1.5		1.1	0.6	1.9		1.0	0.5	2.1		1.1	0.5	2.3		1.9	0.7	5.3	
Enjoy smoking too much		1.3	0.9	1.9		1.2	0.9	1.6		1.0	0.7	1.4		0.9	0.6	1.4		0.8	0.4	1.4	
Smoking ban at home		1.3	0.9	1.9		1.1	0.7	1.8		1.0	0.6	1.6		1.0	0.6	1.8		1.0	0.4	2.3	
*- Log likelihood*		*912.1*				*599.5*				*389.4*				*380.3*				*156.6*			
*N*		*1489*				*1089*				*623*				*1046*				*260*			

NB. *, **, and *** Significant at 0.05, 0.01, and 0.001, respectively; CI, confidence intervals; Odds ratios adjusted for all other variables in the table.

Smokers who used hand-rolled cigarettes, either predominantly or solely, relapsed more in the early period (only significant for first month), but there was no such effect beyond six months.

Several variables reversed their predictive relationships as time quit increased. Higher perceived addiction predicted short-term relapse, but, beyond six months, the direction of effect changed and it was now predictive of abstinence.

Planning to quit within the next month at Wave 1 is a significant predictor of quitting for the first six months, most clearly between one and six months, but was associated with relapse beyond that.

Higher self-efficacy was a strong positive predictor only for short-term quitting, but moderate self-efficacy (somewhat sure) became predictive of relapse beyond one month, while higher levels of self-efficacy trended that way.

Sex, education, region, having a smoking ban at home, and our measures of quitting motivation (perceived benefit if quit, level of worry about health, opinion in their enjoyment of smoking) were all unrelated to abstinence in our analyses.

## 4. Discussion

This is the first large population-based study of the determinants of longer-term quitting outcomes, of which we are aware of, outside of Western countries. The finding that nearly all smokers in our cohort reported at least one quit attempt over the period of the study is quite remarkable. This reflects much more quitting activity than that found in the West [1]. We think the effects are real, and not one of social desirability leading to reporting attempts where none occurred, as reporting attempts led to further questioning, which was generally answered in consistent ways. We think the extraordinary levels of quitting activity is in large part due to a comprehensive package of restrictions and policies, which included the first nation-wide media campaign designed to encourage quitting. Other key tobacco control initiatives over this period include the introduction of graphic health warnings on cigarette packs, tightening of tobacco promotion, increased smoke-free places, and the highly revered king making extensively publicized statements discouraging smoking. We would be surprised if this level of quitting activity persisted. What the implications of this are for success rates of attempts is unclear.

Some of our findings are consistent with research in the West. In particular, being older is associated with successful quitting, consistent with previous studies (e.g., [6,8,16]). We found this association was stronger the longer the period of quitting examined. The positive effect of being in an old age on staying quit is also consistent with Li *et al.* [16] analysis in Southeast Asian context (Malaysia and Thailand). This may be because older people are more likely to experience health problems, and thus are more motivated to stay quit.

We found mixed evidence of success in quitting being reduced as levels of addiction increase. Higher number of cigarettes per day was associated with relapse. This is consistent with other studies [4,10,16,17], most clearly between one and six months, which is a different pattern to that recently found by Yong *et al.* [18] in the ITC 4-country study, which found it to only be predictive in the first month of an attempt. Further, unlike other studies [17,25] we found no association with time to first cigarette, but this may be due to lack of validity of our novel measure rather than reflecting a lack of relationship.

Consistent with past research [4,11], we also found that those who had quit for more than one week previously were most likely to succeed, with most of the effect being on early abstinence. This is a stronger effect than recently found in our ITC 4-country study [26]. Our findings are different from Li *et al.*’s study [16], which finds only the significant effect of previous attempt on making subsequent quit attempt, but not on staying quit in the subsequent follow up.

The finding on self-assessed level of addiction is of considerable interest. This measure predicted early relapse, but surprisingly was associated with abstinence beyond six months. Perhaps those who saw themselves as addicted are more motivated to stay stopped if they can survive the first month.

Our findings on self-efficacy are in line with Li *et al.*’s study [16], which found a predictive effect of higher-level self-efficacy predicting being quit at the next follow-up among smokers in Malaysia and Thailand, similar to our finding of effects on short term outcomes. However, with our findings, self-efficacy reversed its prediction over time quit with high baseline self-efficacy being protective early on, but at least moderate self-efficacy was associated with relapse beyond six months. This may be because those who feel that quitting is easy are more prepared to return back to smoking after starting a quit attempt, based on the thinking that when they need to quit again they will be able to. Less consistent with this, is the failure to find any effects for positive reasons for quitting or for positive reasons for continuing to smoke. The loss of predictive power may also be because self-efficacy changes and by one month quit is essentially unrelated to pre-quit levels, although this does not explain the negative findings.

We found more immediate quitting intention (plan to quit within next month) were predictive of success early on (staying quit for at least six months), consistent with several past studies (e.g., [4,8,16], but the positive effect does not appear to persist beyond six months.

One important null finding was that we found no association with socio-economic characteristics (using education as a proxy measure) significantly predicting successful quitting as found in previous studies (e.g., [9,15]. Other demographic characteristics, which are found predictive of being quit in a previous study that did not show a significant effect in our study, were urban residence (Li *et al.*, 2010) and being female [15]. Demographic and socio-economic status may not be a major barrier to successful quitting in Thailand.

As in Borland *et al.*’s [1] study, where they found that, in their four Western countries, a majority of smokers have succeeded in staying quit for more than one month and around one-third have stopped for more than six months, this study shows that Thai smokers are at least as capable of achieving short and medium term goals, but, like in the West, long-term relapse remains a problem.

The strengths of this study include its prospective cohort design, large sample size and long follow-up period over four years. A limitation of our study is the use of self-reported data to determine smoking outcomes and quit length, which may be subject to recall and social desirability biases. Given that this is a non-intervention study, it is unlikely that respondents would lie about their smoking status. Past reviews of population-based survey studies have confirmed the accuracy of self-report, and conclude that biochemical validation of smoking status is unnecessary [27,28]. Another study limitation is the potential threat to external validity of the study due to sample attrition. Our ability to find predictors for the long-term quitting outcomes is reduced by the necessarily smaller samples available for analysis. That said, attrition here was remarkably small, and we have no reason to believe there would be different determinants of relapse for those who dropped out of our study.

We also note that we used baseline measures to predict outcomes, and in some cases would have updated information from closer to the index attempts. Use of these measures may have improved prediction, but we think it unlikely to have affected the patterns of changing prediction. We considered using the most recent predictor data, but because in some cases the predictors would need to change from one outcome to another, decided it added undue complexity to an already complex study.

## 5. Conclusions

In conclusion, nearly all Thai smokers tried to quit over the four year period studied, and, while most can survive a month, subsequent relapse is common. The determinants of relapse appear to change with time quit, but in somewhat different ways to patterns found in the West. In particular, heavy cigarette consumption appears to predict relapse over a more sustained period in Thailand. Preventing longer-term relapse must be a priority if smoking rates are to be reduced more rapidly. More study is required of longer-term relapse, as most existing studies are dominated by short-term relapse. Determinants of quitting in countries with different tobacco control histories may differ and differences require further study. Even given the high rates of relapse, there was plenty of sustained quitting, which provides a basis for smoking prevalence in Thailand declining.
